# Reflex Irritations and Neuroses Caused by Stricture of the Urethra in the Female

**Published:** 1892-04

**Authors:** Fessenden N. Otis


### REFLEX IRRITATIONS AND NEUROSES CAUSED BY STICTURE OF THE URETHRA IN THE FEMALE.

BY FESSENDEN N. OTIS, M. D.

*Medical Record*, New York, 1892, xli., No. 2.

In this article the author advances the fact that stricture of the urethra in the female has been but little considered either in works on special diseases of the female genito-urinary organs, or in those solely devoted to the subject of urethral stricture, and hence it might be inferred to be a rare and unimportant difficulty. At the same time, symptoms occurring in the female which in the male would at once suggest the probable presence of urethral stricture, are referred to as "irritable bladder," and are attributed to causes quite independent of their possible relations to the urethra. Although over-distention in such cases is one of the most efficacious means of treatment, " examination of the urethral canal for conditions which would justify such procedure seems to have been completely lost sight of."

Organic urethral stricture in either the male or the female, in the opinion of the author, is *inevitably* due to the exudation and organization of plastic lymph in the subepithelial urethral tissues, any irritation sufficient to cause this producing stricture, though the inflammatory process may be of so low a grade as to escape the cognizance of the subject of it. Strictures are formed in this way, from the irritation of the urine, in subjects of gouty or rheumatic diatheses, practically identical with those resulting from gonorrhœa, and the foundations, at least, of the largest proportion of urethral strictures in the male are due to plastic deposits from attacks of lithiasis " often long antecedent to the gonorrhœas to which they are attributed."

After a brief reference to frequent and severe reflexes occurring in the male caused by strictures of even large calibre (brought prominently before the profession in 1874), Prof. Otis presents a series of cases to demonstrate that stricture of the urethra may occur in the female sufficient to cause reflex symptoms equally severe, and also that in cases where relief is not obtained from ordinary measures, the urethra should be explored by means of the urethrometer or bulbous sound, if only to eliminate an important element in the diagnosis and treatment of these cases.

In the treatment of these cases of stricture of the urethra in the female, divulsion is probably a much less serious operation than in the male, and, when carried to an extreme degree, rarely results in a permanent loss of control of the sphincter. The tendency to recontraction is marked, however, and in most instances the contractions will yield to gradual dilatation. The symptoms having once been relieved, the calibre of the canal may be maintained by the patient herself introducing the closed urethrometer into the bladder, expanding it to the degree required, and then gently withdrawing it.—*International Medical Magazine*, March, '92, p. 198.

